# Aeroallergen Sensitization Patterns and Related Factors in Children With Allergic Rhinitis in Guangzhou

**DOI:** 10.1155/mi/5887915

**Published:** 2025-02-25

**Authors:** Qingxiang Zeng, Chao Yang, Jinyuan Li, Xiangqian Qiu, Wenlong Liu

**Affiliations:** Department of Otolaryngology, Guangzhou Women and Children's Medical Center, Guangzhou Medical University, Guangzhou 510623, China

**Keywords:** aeroallergen sensitization, aeroallergens, allergic rhinitis, children, COVID-19

## Abstract

**Background:** Certain patterns of children's serum immunoglobulin E (IgE) sensitivity to aeroallergens may offer useful clinical insight into forecasting the course and prognosis of allergic rhinitis (AR). The study aimed to investigate the changes in aeroallergen sensitization patterns in children with AR during the last decade and compare the sensitization pre- and post-coronavirus disease 2019 (COVID-19) pandemic in Children who visited our center.

**Methods:** This is a retrospective study, examining the serum IgE of nine aeroallergens from 21,362 children (1–12 years old) from AR who visited Guangzhou Women and Children's Medical Center from June 2013 to June 2023.

**Result:** The dust mites were the most prevalent aeroallergen in Guangzhou, with positive sensitization rates of 74.30% for *Dermatophagoides farinae* (*D. farinae*), 73.30% for *Dermatophagoides pteronyssinus* (*D. pteronyssinus*), and common ragweed (1.6%) was the lowest. After the COVID-19 pandemic, the sensitization rates to *D. farinae* were consistent and *D. pteronyssinus* was slightly decreased while German cockroach, cat, and dog dander were increased. Most of the aeroallergens other than common ragweed were increased in school-age children than preschool stage. Boys have a higher positive rate than girls for *D. farinae* and *D. pteronyssinus*.

**Conclusions:** With the unraveling of allergens' sensitization rates in various conditions, avoidance from *D. farinae* and *D. pteronyssinus* should still be the most important objectives to maintain in reducing AR episodes.

## 1. Introduction

Allergic rhinitis (AR) is a type I allergic disorder characterized by symptoms of nasal hyper-reactivity, such as sneezing, nasal pruritus, and airflow restriction. One recognized risk factor for allergic illnesses is atopy, which is defined as the genetic predisposition to produce immunoglobulin E (IgE) antibodies in response to allergen exposure [1]. IgE-antibody reactions to environmental allergens demonstrate that “molecular spreading” occurs in early infancy and predates the onset of clinical illness. Exposure to indoor inhalant allergens increases the chance of specific sensitivity, even developing asthma, and reduced lung function in children [2].

In mainland China, a recent 4-year multicenter study found that the prevalence characteristics of allergen sensitization varied among patients with allergic symptoms by gender, age, place of residence, and season, and these differences could be caused by a variety of factors, such as cross-reactivity, lifestyle choices, socioeconomic status, genetic predispositions, and climate changes [3].

According to a study published in 2016, which investigated the prevalence and trends of sensitization to aeroallergens in patients with AR in Guangzhou, the most prevalent aeroallergen was house dust mites (HDMs) [4]. When patients exhibiting nasal hyper-responsiveness were categorized based on their age in decades, an upward trend in age correlated with a roughly 5.13% decline in the positivity rate of specific IgE (sIgE) [4]. They found that sensitization to pet allergens has been more common in 2014 than in 2005; thus, for young children with AR, pet allergies should be prioritized in light of the growing trend of sensitization to cat and dog dander [4, 5]. During the coronavirus disease 2019 (COVID-19) pandemic, sensitivity rates in school-age children with AR increased for specific allergens such as HDMs, cat, pollen, *Artemisia*, and *Cupressus* in Turkey [6]. Another study found that atopic patients experienced an elevation in the prevalence of allergies to cats and dogs throughout the COVID-19 outbreak period [7]. Decreased exposure to the environment, changed lifestyles, and biodiversity loss all contribute to a less diverse microbiome, which is linked to allergic reactions [8].

In order to further assess the sensitization trends in Guangzhou in early life, we focused on AR children to explore the specific patterns in the new decade.

## 2. Materials and Methods

### 2.1. Study Design and Clinical Data Acquisition

This was a 10-year retrospective study (January, 2014 to December, 2023) conducted on 21,632 children with AR. Patients enrolled before January 25, 2020 (the start of lockdown measures in Guangzhou), including those enrolled on that day, are defined as pre-COVID-19 pandemic, while patients enrolled after January 25, 2020, are defined as post-COVID-19 pandemic. Children were diagnosed with AR (at least one type of positive allergen) by clinical specialists. All participants in the study population met the inclusion criteria for AR [9], which necessitated a minimum of 24 months of disease duration (typical symptoms, including rhinorrhea, nasal obstruction, itching, and sneezing) and evidence of sensitization through positive sIgE levels against at least one prevalent aeroallergen. Furthermore, subjects were excluded if they had a history of other allergic diseases such as asthma (excluded using spirometric measurements) or atopic dermatitis, severe systemic, immune, or psychological disorders. Data on gender, age, outpatient visited date, and family locations were gathered from electronic medical record files for further analysis. The protocol was approved by the Ethics Committee of Guangzhou Women and Children's Medical Center, Guangzhou Medical University (261A01). Written informed consents were obtained from guardians of children in this study.

### 2.2. Allergen-Specific sIgE Detection

Serum samples from AR children were gathered and detected. The ImmunoCAP 1000 system (Thermo Fisher Scientific, USA), a fully automated in vitro allergen detector, was utilized to detect allergen-specific sIgE in the sera. The sIgE levels for nine categories of aeroallergens were evaluated, including those for the HDM: *Dermatophagoides farinae* (*D. farina*, d1), *Dermatophagoides pteronyssinus* (*D. pteronyssinus*, d2), German cockroach (i6, Blatella germanica), cat dander (e1), dog dander (e5), common ragweed (Amb a1), mugwort (Art v), weed pollen (tx4: *Quercus alba*, *Ulmus americana*, *Platanus acerifolia*, *Salix caprea*, *Populus deltoides*), mold mix (mx1: *Penicillium chrysogenum*, *Cladosporium herbarum*, *Aspergillus fumigatus*, *Alternaria alternata*). A serum sIgE level ≥0.35 kUA/L was defined as positive.

### 2.3. Statistical Analysis

Statistical analysis was performed using the SPSS 20 software. The data are presented as the median, discrete numbers, and percentages. The Chi-square or Fisher's exact test was utilized for comparisons. A value of *p* < 0.05 was considered statistically significant.

## 3. Results

### 3.1. Overall Prevalence of Aeroallergen Sensitization of Children in Guangzhou

The demographic information is summarized in Table 1. Among the 21,632 AR children, the prevalence rates of sensitization to aeroallergens were as follows: 74.30% for *D. farinae*, 73.30% for *D. pteronyssinus*, which were the highest positive sensitization rate within those most common aeroallergens, and 5.60% for cockroaches, 5.50% for cat dander, 3.80% for dog dander, 3.6% for mugwort, 3.1% for weed pollen, 4.6% for mold mix, and 1.6% for common ragweed, which was the lowest. The monosensitization rate in the AR children was 4.5%, and the polysensitization rate was 95.5%, but when *D. farina* and *D. pteronyssinus* were considered one type sensitization, the monosensitization rate became 52.6%.

### 3.2. Aeroallergen Sensitization by Age Groups

To further assess the impact of age on the prevalence of allergic sensitization among AR children, we grouped them as follows: preschool children (1–6 years), school-age children (7–12 years), and youngsters (13–18 years).

Table 2 shows that the positive rates of sensitization to common ragweed in preschool children, school-age children, and youngsters were 1.50%, 1.60%, and 2.30% without significant differences.

Nevertheless, other than common ragweed, the sensitization positive rates of the other aeroallergens tested were increased in a school-age group than in preschool children. The positive rates of *D. farinae*, *D. pteronyssinus*, cockroaches, cat dander, and mold mix were increased from 67.80%, 67.10%, 4.20%, 4.00%, 3.6% in preschool children to 81.80%, 80.40%, 7.00%, 7.20%, 6.00% in school-age children, and were even higher in kept in the youngsters as 82%, 80.90%, 8.90%, 9.40%, and 8.70% (all *p* < 0.0001).

As to the dog dander, mugwort, and weed pollen, the positive rates were also higher in school-age children than in preschool children (all *p* < 0.0001), but there was no difference between youngsters and school-age or preschool children (Table 2).

### 3.3. Aeroallergen Sensitization by Gender

As shown in Table 3, the prevalence between male (M) and female (F) is different with sensitization to *D. farinae* (M:F 76.10%:70.40%), *D. pteronyssinus* (M:F 75.10%:69.50%), cockroaches (M:F 6.00%:4.70%), and weed pollen (M:F 3.80%:2.00%). Interestingly, with the above four aeroallergens, the positive rate was all higher in males than in females (all *p* < 0.0001).

However, there is no difference between males and females with the sensitization to cat dander (M:F 5.60%:5.50%), dog dander (M:F 3.80%:4.00%), common ragweed (M:F 1.70%:1.30%), mugwort (M:F 3.60%:3.60%), and mold mix (M:F 4.70%:4.20%).

Then, we further divided the males and females into different age groups following the same rules as above (Table 4). First of all, in preschool children, the positive rates of *D. farinae* (M:F 69.50%:64.00%), *D. pteronyssinus* (M:F 75.10%:69.50%), cockroaches (M:F 6.00%:4.70%) were higher in male (all *p* < 0.0001). Second, in school-age children, other than the above three allergens, weed pollen was also higher in males than in females (M:F 5.20%:1.80%) (*p* < 0.0001). Lastly, only the positive rates of *D. farinae* (M:F 84.40%:76.90%) and *D. pteronyssinus* (M:F 84.60%:74.80%) were higher in male youngsters (all *p* < 0.0001).

### 3.4. Aeroallergen Sensitization by Geographic Districts of Guangzhou City

Guangzhou is the provincial capital of Guangdong. We further analyzed the difference in 11 municipal districts (Figure S1), as shown in Table 5. Sensitizations to *D. farina*, cat dander, and dog dander were highest in the Yuexiu district (79.10%, 10.30%, and 5.90%, respectively) (all *p* < 0.0001). Cockroaches and weed pollen were highest in the Conghua district (9.6% and 7.4%) (all *p* < 0.0001). Sensitization to the other allergens showed no difference among different municipal districts, as shown in Table 5.

According to longitude and latitude, there are four different coordinate areas, Conghua, Huadu and Zengcheng, Panyu and Nansha, and other districts. Within the aeroallergens tested, only cockroaches and weed pollen showed different positive sensitization rates in different coordinates, both of which had the highest positive rate in the longitude and latitude where the Conghua district is located (all *p* < 0.0001).

According to the different precipitation, we divided Guangzhou into three districts: the highest precipitation areas (>2500 mm/year), which consist of Conghua and Zengcheng districts; the lowest precipitation areas (<1500 mm/year), which consist of Panyu and Nansha districts, Huadu and the left other districts were in the middle as 2000–2300 mm/year. Cockroaches and weed pollen showed a higher positive rate with higher precipitation in the Conghua and Zengcheng districts than Panyu and Nansha districts, Huadu, and the left other districts (all *p* < 0.005). While the positive rate of dog dander in the Conghua and Zengcheng districts was lower than in Huadu and the other districts (all *p* < 0.005).

### 3.5. Aeroallergen Sensitization Before and After COVID-19 Pandemic

With the pandemic of COVID-19, detergents, disinfectants and other hygiene activities were massively used in daily life. Upon data analysis, we discovered that the rates of positive responses to *D. farinae* and weed pollen were consistent both before and after the COVID-19 pandemic.

However, there was a slightly decrease in positive rate from 74.40% to 72.50% in *D. pteronyssinus* and an obvious decrease from 7.60% to 4.4% in mold mix (Table 6). Meanwhile, the positive sensitization rate of cockroaches (3.80% vs. 6.70%), cat dander (1.80% vs. 8.10%), dog dander (1.00% vs. 5.80%), common ragweed (0.70% vs. 2.50%), and mugwort (0.50% vs. 6.60%) were all increased after COVID-19 pandemic (all *p* < 0.05).

### 3.6. Aeroallergen Sensitization Among Gross Domestic Product (GDP) Levels of Municipal Districts

We use the GDP level per capita to divide the districts of the city into four levels. Highly developed areas (>300,000 yuan), including Yuexiu and Huangpu districts; moderately developed areas, including Tianhe and Nansha districts; less developed areas, including Huazhu, Huadu, and Panyu districts; and undeveloped areas, including Liwan, Zengcheng, Baiyun, and Conghua districts. To our surprise, as shown in Table 7, the positive sensitization rate to cockroaches in the highly developed areas (5.00%) was almost as high as that in the undeveloped areas (5.2%), greater than that in the moderately developed areas (3.50%) and the less developed areas (4.4%) (*p* < 0.005). What is more, the positive sensitization rates to cat dander were also showed the same trend, the highest in the highly developed areas (7.0%) and the undeveloped areas (6.6%), lowest in the less developed areas (4.4%) (*p* < 0.005). The other aeroallergens tested were no difference in the positive rate according to GDP level.

## 4. Discussion

In our study, we presented here a relatively comprehensive distribution of the inhalant allergens' sensitization in children with AR in different settings in Guangzhou and the changes after the COVID-19 pandemic.

The prevalence patterns of aeroallergens in Guangzhou remained the highest for *D. farinae* and *D. pteronyssinus*. Due to the warm and humid climate, HDMs were the most common allergen of airway allergic inflammation, including asthma and AR, in southern China, while pollen constituted the majority in northern China [10]. By adopting latent class analysis (LCA), researchers found that HDMs groups were prevalent not only in the south but also in the east of China, whereas pollen sensitization clusters were primarily found in the country's north or west [11]. More specifically, an investigation carried out within the Greater Bay Area (GBA) of Guangdong, Hong Kong, and Macao revealed a correlation between variations in the weather and the quantity of outpatient visits to AR. It has been demonstrated that the number of outpatient visits for AR may be correlated with different climates, such as low temperature, low humidity, and high wind speed [12].

The prevention, screening, diagnosis, and treatment of allergies may be significantly impacted by the differences in age, gender identity, and their relationship to allergen sensitizations. The current study showed that the positive sensitization rates to indoor aeroallergens, including *D. farinae*, *D. pteronyssinus*, cockroaches, cat dander, and mold mix, were significantly higher in school-age children and youngsters than in preschool children. The result was in line with the previous study, which found that the prevalence of HDM, German cockroach, increases steadily with age before and during the teenage years [3].

A study showed in children of 0–10 years old, boys are more likely than girls to have AR. Conversely, compared to their male counterparts, females exhibit a greater frequency of AR during adolescence of 11–17 years [13]. A few meta-analyses indicated that sex-related differences existed in allergy prevalence with a switch at around puberty, from male predominance to female [14, 15]. Our study demonstrated that the predominance might differ with regard to different allergens. In the entire childhood period, the sensitization rate of dust mites was higher in males than in females, while cockroaches had the same trend in preschool and school stages, but the difference between sex lost in youngsters. As to the weed pollen, school-age boys had a higher positive rate than girls but not in the other stages. Sexual hormones, gender-specific lifestyle choices, dietary differences, microbiome diversity, and treatment compliance are a few possible causes [13].

According to reports, China was exposed to the novel coronavirus (SARS-CoV-2). The Chinese people adopted preventive precautions, like wearing properly fitted face masks, cleaning hands frequently with alcohol-based hand rub, and keeping a safe distance from others in response to the internal and international spread of COVID-19. However, strict hygiene practices might not always be advantageous for the immune system. Several investigations, including the famous “Hygiene Hypothesis,” have demonstrated that early life exposure to a low diversity of microbes raises IgE levels, which in turn increases the probability of developing allergic diseases [6]. However, results from a study demonstrated that during the COVID-19 outbreak from 2020 to 2021, maintaining good hygiene reduced the risk of infection, which could account for the declines in IgG levels in both adults and children, but IgE levels did not change from the pre-COVID-19 which was before 2019 to the COVID-19 period [16]. In our present study, we found that after almost 3 years of strict hygiene regulations, there was a slightly decrease in serum IgE sensitization rate to *D. pteronyssinus* and an obvious decrease in mold mix. Meanwhile, the positive sensitization rates of cockroaches, cat, and dog dander were significantly increased. The only study on changes in allergens among adults before and after the COVID-19 pandemic reveals that a significant number of individuals have developed sensitivities to cats or dogs. This increase in sensitivity is attributed to heightened exposure to pet antigens, both directly and indirectly, during the COVID-19 epidemic [7]. To our surprise, with regard to the outdoor inhalant allergens, the sensitization rates to common ragweed and mugwort were all increased after the COVID-19 pandemic. Whether it is due to the large-scale manipulation of the environment over the pandemic period needs further investigation. On the contrary, Liu's study found that there was an elevation in the incidence of sensitization to indoor airborne allergens, whereas no notable alterations were discernible in the sensitization to outdoor airborne allergens in comparison to the sensitization rates observed 2 years prior to the emergence of the COVID-19 pandemic [17]. Zhang's team investigated the distribution and pattern of aeroallergens in Sichuan, China, after the COVID-19 epidemic and the significant reduction in allergy prevalence compared to the pre-epidemic period [18]. Sule's report suggested that there was an increase in the frequency of sensitization to cats, the rates of cat ownership, and the severity of cat-induced allergic symptoms throughout the COVID-19 pandemic [19]. The differences in the pattern of aeroallergens in different regions may be attributed to geographical conditions, climate and temperature, and genetic factors.

The reasons for the changes in allergens following COVID-19 are complex and multifaceted. We believe they may be involved in the following mechanisms: (1) The incidence of upper respiratory tract infections and exposure to outdoor allergens and contaminants decreased significantly during the COVID-19 pandemic, while it has been documented that viral respiratory pathogens, such as rhinoviruses and respiratory syncytial viruses, augment the induction of airway epithelia to secrete pro-T helper 2 cytokines, namely TSLP, IL-25, and IL-33, which facilitate the occurrence of sensitization [20]. (2) Higher concentrations of NO_2_, CO, and PM10 before and after the COVID-19 lockdowns compared to during the lockdown period, while exposure to hazardous air pollutants can impair airway function [21]. (3) The mechanisms by which COVID-19 impacts AR remain unclear, with varying reports in the literature. In the context of SARS-CoV-2 infection, a significant reduction in angiotensin-converting enzyme 2 (ACE2) expression and an increase in transmembrane protease serine 2 (TMPRSS2) expression have been observed not only in the lower airway epithelial cells but also in the nasal epithelial cells of patients with asthma and AR. Based on these findings, the decreased ACE2 expression in patients with AR may confer a protective effect against COVID-19. Conversely, another study indicates that ACE2 expression in nasal tissues is unaffected in AR, as ACE2 expression correlates with the interferon response rather than the type 2 response in nasal tissues and epithelial cells [22].

Several limitations should be taken into consideration when interpreting our findings. First, we had only collected data in the childhood stage and it would be essential to conduct longitudinal research with follow-up continuing into adulthood in the future. Second, allergenic components such *Der p 23* have been shown to be associated with AR symptoms and have more precisely predictive value of the effect of allergen immunotherapy [23, 24], but we were unable to detect the exact components of aeroallergens. Third, the severity of symptoms was not collected so the lack of relationship between disease severity and serum sIgE level is another limitation of our study. Fourth, multicenter researches similar to our study would enhance the applicability of the findings to a broader context. Fifth, a thorough examination in subsequent research, employing advanced statistical techniques such as multiple regression analysis or alternative multivariate methodologies, could offer more profound understanding of the interplay and cumulative effects of variables, including age, gender, geographical location, and the pandemic on sensitization prevalence. Sixth, although it would be more meaningful to detect changes in allergens for the same group of AR patients before and after the pandemic, this is almost impossible to achieve. Therefore, we can only detect changes in allergens for pediatric AR patients in the same region before and after the pandemic.

## 5. Conclusion

Hygienic behaviors during the COVID-19 pandemic did not lead to an increase of the positive sensitization rate to *D. farinae* and *D. pteronyssinus*, while German cockroach, cat dander, and dog dander were increased. Avoidance from *D. farinae* and *D. pteronyssinus* should still be the most important objectives to maintain in reducing AR episodes. Moreover, the provision of actionable suggestions for tailoring allergen screening methodologies according to geographically distinct and age-stratified sensitization profiles would offer greater utility. Furthermore, offering directives on the incorporation of these insights into routine management strategies for pediatric AR would augment the clinical applicability of the research outcomes in practical settings.

## Figures and Tables

**Table 1:** Demographic information of AR children.

Characteristics	
Cases	21,362
Sex ratio (male:female)	2.04:1
Age	6.8 ± 2.5
Living environment (urban versus rural residence)	6.9:1
Family history of allergy	12,375 (57.9)
Aeroallergen sensitization	
*D. farinae*	74.3%
*D. pteronyssinus*	73.3%
German cockroach	5.6%
Cat dander	5.5%
Dog dander	3.8%
Common ragweed	1.6%
Mugwort	3.6%
Weed pollen	3.1%
Mold mix	4.6%

Abbreviation: AR, allergic rhinitis.

**Table 2:** Aeroallergen sensitization of AR children by age groups.

Aeroallergens	1–6 years	7–12 years	13–18 years	*p*
*D. farinae*	7859 (67.8%)	7673 (81.8%)*⁣*^*∗*^	523 (82.0%)*⁣*^*∗*^	**<0.0001**
*D. pteronyssinus*	7775 (67.1%)	7547 (80.4%)*⁣*^*∗*^	519 (80.9%)*⁣*^*∗*^	**<0.0001**
German cockroach	485 (4.2%)	651 (7.0%)*⁣*^*∗*^	57 (8.9%)*⁣*^*∗*^	**<0.0001**
Cat dander	461 (4.0%)	668 (7.2%)*⁣*^*∗*^	60 (9.4%)*⁣*^*∗*^	**<0.0001**
Dog dander	347 (3.0%)	445 (4.8%)*⁣*^*∗*^	29 (4.6%)	**<0.0001**
Common ragweed	129 (1.5%)	123 (1.6%)	12 (2.3%)	0.3372
Mugwort	268 (3.1%)	318 (4.1%)*⁣*^*∗*^	19 (3.6%)	**0.0013**
Weed pollen	68 (2.4%)	72 (4.2%)*⁣*^*∗*^	8 (7.0%)	**0.0001**
Mold mix	102 (3.6%)	102 (6.0%)*⁣*^*∗*^	10 (8.7%)*⁣*^*∗*^	**<0.0001**

*Note:* Bold for significant differences.

Abbreviation: AR, allergic rhinitis.

*⁣* ^*∗*^Compared with 1–6 years group, *p* < 0.05 by Chi-square test.

**Table 3:** Aeroallergen sensitization of AR children by gender.

Aeroallergens	Male	Female	*p*
*D. farinae*	11,053 (76.1%)	5002 (70.4%)	**<0.0001**
*D. pteronyssinus*	10,908 (75.1%)	4933 (69.5%)	**<0.0001**
German cockroach	866 (6.0%)	327 (4.7%)	**<0.0001**
Cat dander	802 (5.6%)	387 (5.5%)	0.8489
Dog dander	543 (3.8%)	278 (4.0%)	0.5445
Common ragweed	191 (1.7%)	73 (1.3%)	0.0854
Mugwort	405 (3.6%)	200 (3.6%)	0.86
Weed pollen	118 (3.8%)	30 (2.0%)	**0.0005**
Mold mix	148 (4.7%)	66 (4.2%)	0.4585

*Note:* Bold for significant differences. Data were analyzed by Chi-square test.

Abbreviation: AR, allergic rhinitis.

**Table 4:** Aeroallergen sensitization of AR children by gender and age.

Aeroallergens	1–6 years		*p*	7–12 years		*p*	13–18 years		
	M	F		M	F		M	F	*p*
*D. farinae*	5278 (69.5%)	2581 (64%)	**＜0.0001**	5441 (83.4%)	2232 (78%)	**＜0.0001**	334 (84.4%)	189 (76.9%)	**0.0213**
*D. pteronyssinus*	5230 (68.8%)	2545 (63.7%)	**＜0.0001**	5343 (81.9%)	2204 (77%)	**＜0.0001**	335 (84.6%)	184 (74.8%)	**0.0027**
German cockroach	340 (4.5%)	145 (3.7%)	**0.0317**	493 (7.6%)	158 (5.6%)	**0.0003**	33 (8.4%)	24 (9.8%)	0.5694
Cat dander	302 (4%)	159 (4%)	＞0.9999	469 (7.2%%)	199 (7%)	0.695	31 (7.9%)	29 (11.8%)	0.0964
Dog dander	221 (3%)	126 (3.2%)	0.4565	309 (4.8%)	136 (4.8%)	＞0.9999	13 (3.3%)	16 (6.6%)	0.0769
Common ragweed	91 (5632%)	38 (1.3%)	0.3034	94 (1.8%)	29 (1.2%)	0.0937	6 (1.8%)	6 (3%)	0.385
Mugwort	181 (3.2%)	87 (2.9%)	0.6008	212 (4%)	106 (4.5%)	0.3209	12 (3.7%)	7 (3.5%)	＞0.9999
Weed pollen	50 (2.7%)	18 (1.8%)	0.1256	63 (5.2%)	9 (1.8%)	**0.0008**	5 (7.2%)	3 (6.5%)	＞0.9999
Mold mix	68 (3.7%)	34 (3.3%)	0.7519	74 (6.1%)	28 (5.6%)	0.737	6 (8.7%)	4 (8.7%)	＞0.9999

*Note:* Bold for significant differences. Data were analyzed by Chi-square test.

Abbreviations: AR, allergic rhinitis; F, female; M, male.

**Table 5:** Aeroallergen sensitization of AR children by geographic districts of Guangzhou City.

Aeroallergens	BY	CH	PY	HZ	HD	HP	LW	NS	TH	YX	ZC	*p*
Df	2017 (73.1%)	326 (74.3%)	997 (73.1%)	1339 (76.3%)	832 (73.5%)	992 (73.2%)	962 (75.7%)	260 (77.4%)	2192 (74.7%)	983 (79.1%)	1157 (75.4%)	0.0043
Dp	1989 (72.1%)	332 (76%)	985 (72.3%)	1303 (74.2%)	812 (71.7%)	995 (73.4%)	936 (73.6%)	252 (75.0%)	2162 (73.6%)	943 (75.9%)	1147 (74.7%)	0.2399
Cockroach	146 (5.3%)	42 (9.6%)	66 (4.9%)	58 (3.4%)	68 (6.1%)	69 (5.1%)	56 (4.5%)	12 (3.6%)	102 (3.5%)	59 (4.8%)	90 (5.9%)	**<0.0001**
Cat	130 (4.8%)	22 (5.1%)	85 (6.3%)	145 (8.3%)	72 (6.4%)	52 (3.9%)	90 (7.1%)	19 (5.7%)	151 (5.2%)	128 (10.3%)	75 (4.9%)	**<0.0001**
Dog	102 (3.7%)	11 (2.6%)	55 (4.1%)	90 (5.2%)	45 (4.0%)	41 (3.1%)	59 (4.7%)	10 (3.0%)	125 (4.3%)	73 (5.9%)	47 (3.1%)	**0.0009**
Ragweed	36 (1.7%)	7 (2.0%)	18 (2%)	15 (1.1%)	17 (2.0%)	19 (1.7%)	14 (1.4%)	8 (3.0%)	35 (1.5%)	16 (1.7%)	28 (2.2%)	0.4194
Mugwort	76 (3.6%)	12 (3.5%)	38 (4%)	47 (3.3%)	36 (4.3%)	52 (4.6%)	32 (3.3%)	19 (7.2%)	85 (3.6%)	36 (3.8%)	45 (3.6%)	0.2017
Pollen	17 (2.6%)	7 (7.4%)	4 (1.4%)	5 (1.4%)	6 (2.1%)	11 (4.8%)	10 (3.3%)	4 (5.6%)	13 (2.3%)	11 (3.6%)	17 (6.0%)	**0.0031**
Mold mix	33 (5.0%)	1 (1.1%)	14 (5.1%)	7 (2.0%)	11 (3.8%)	10 (4.4%)	13 (4.3%)	2 (2.8%)	25 (4.4%)	11 (3.6%)	12 (4.2%)	0.5309

*Note:* Bold for significant differences. Data were analyzed by Fisher's exact test.

Abbreviations: AR, allergic rhinitis; BY, Baiyun district; CH, Conghua district; *Df*, *D. farina; Dp*, *D. pteronyssinus;* HD, Huadu district; HP, Huangpu district; HZ, Haizhu district; LW, Liwan district; NS, Nansha district; PY, Panyu district; TH, Tianhe district; YX, Yuexiu district; ZC, Zengcheng district.

**Table 6:** Aeroallergen sensitization of AR children before and after the COVID-19 pandemic.

Aeroallergens	Before COVID-19	After COVID-19	*p*
*D. farinae*	6504 (74.70%)	9551 (74.00%)	0.2224
*D. pteronyssinus*	6478 (74.40%)	9363 (72.50%)	**0.0019**
German cockroach	331 (3.80%)	862 (6.70%)	**<0.0001**
Cat dander	149 (1.80%)	1040 (8.10%)	**<0.0001**
Dog dander	80 (1.00%)	741 (5.80%)	**<0.0001**
Common ragweed	55 (0.70%)	209 (2.50%)	**<0.0001**
Mugwort	43 (0.50%)	562 (6.60%)	**<0.0001**
Weed pollen	10 (3.50%)	138 (3.10%)	0.7262
Mold mix	21 (7.60%)	193 (4.40%)	**0.0166**

*Note:* Bold for significant differences. Data were analyzed by Chi-square test.

Abbreviations: AR, allergic rhinitis; COVID-19, coronavirus disease 2019.

**Table 7:** Aeroallergen sensitization among GDP levels of municipal districts.

Aeroallergens	HD	MD	LD	UD	*p*
*D. farinae*	1975 (76.0%)	2452 (74.9%)	2171 (75.2%)	4616 (75.3%)	0.7968
*D. pteronyssinus*	1938 (74.6%)	2414 (73.8%)	2115 (73.2%)	4530 (73.9%)	0.7131
German cockroach	128 (5.0%)*⁣*^*∗*^	114 (3.5%)	126 (4.4%)	314 (5.2%)*⁣*^*∗*^	**0.0028**
Cat dander	180 (7.0%)	170 (5.2%)	217 (4.4%)^#^	404 (6.6%)	**0.0019**
Dog dander	114 (4.4%)	135 (4.2%)	135 (4.7%)	252 (4.2%)	0.615
Common ragweed	35 (1.7%)	43 (1.6%)	32 (1.4%)	81 (1.7%)	0.8618
Mugwort	88 (4.30%)	104 (4.0%)	83 (3.7%)	172 (3.6%)	0.5366
Weed pollen	22 (4.1%)	17 (2.6%)	11 (1.7%)	45 (3.4%)	0.0766
Mold mix	21 (4.0%)	27 (4.2%)	18 (2.8%)	44 (3.4%)	0.5363

*Note:* Bold for significant differences. Data were analyzed by Fisher's exact test.

Abbreviations: GDP, gross domestic product; HD, highly developed area; LD, less developed areas; MD, moderately developed areas; UD, undeveloped areas.

*⁣* ^*∗*^Compared with MD group, *p* < 0.05.

^#^Compared with other groups, *p* < 0.05.

## Data Availability

The data that support the findings of this study are available from the corresponding author upon reasonable request.

## Supporting Information

Supporting Information Figure S1. Map of Guangzhou.
