# Supplementary figures and images for: Aeroallergen Sensitization Patterns and Related Factors in Children With Allergic Rhinitis in Guangzhou
Source: Mediators Inflamm. 2025 Feb 25;2025:5887915. doi: 10.1155/mi/5887915 (PMC11879600; doi:10.1155/mi/5887915)

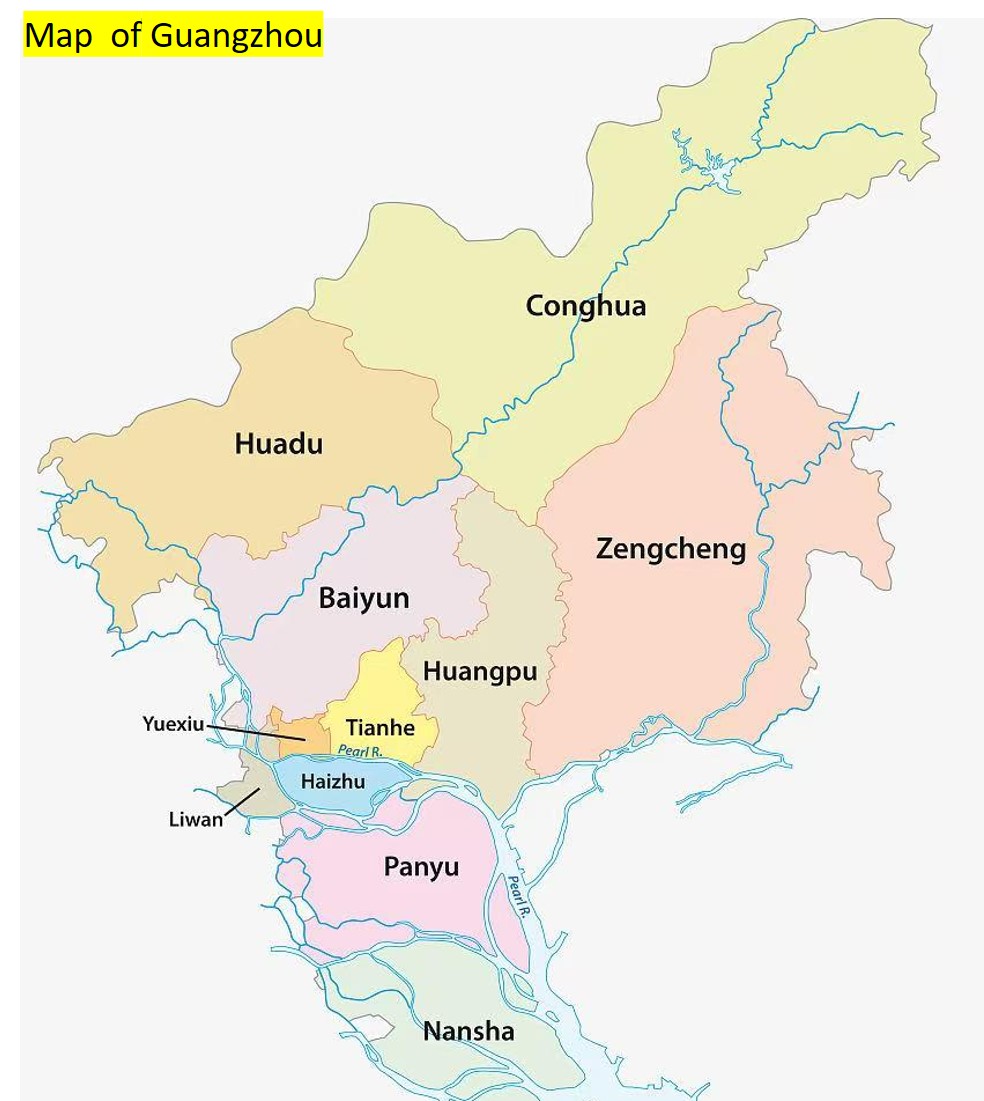

Supplement: Supporting Information — Figure S1. Map of Guangzhou. [file 5887915.f1.jpg]
